# SARS2Mutant: SARS-CoV-2 amino-acid mutation atlas database

**DOI:** 10.1093/nargab/lqad037

**Published:** 2023-04-24

**Authors:** Karim Rahimian, Ehsan Arefian, Bahar Mahdavi, Mohammadamin Mahmanzar, Donna Lee Kuehu, Youping Deng

**Affiliations:** Institute of Biochemistry and Biophysics (IBB), University of Tehran, Tehran, Iran; Department of Microbiology, School of Biology, College of Science, University of Tehran, Tehran, Iran; Department of Computer Science, Tarbiat Modares University, Tehran, Iran; Department of Quantitative Health Sciences, John A. Burns School of Medicine, University of Hawaii at Manoa, Honolulu, HI 96813, USA; Department of Quantitative Health Sciences, John A. Burns School of Medicine, University of Hawaii at Manoa, Honolulu, HI 96813, USA; Department of Quantitative Health Sciences, John A. Burns School of Medicine, University of Hawaii at Manoa, Honolulu, HI 96813, USA

## Abstract

The coronavirus disease 19 (COVID-19) is a highly pathogenic viral infection of the novel severe acute respiratory syndrome coronavirus 2 (SARS-CoV-2), resulted in the global pandemic of 2020. A lack of therapeutic and preventive strategies has quickly posed significant threats to world health. A comprehensive understanding of SARS-CoV-2 evolution and natural selection, how it impacts host interaction, and phenotype symptoms is vital to develop effective strategies against the virus. The SARS2Mutant database (http://sars2mutant.com/) was developed to provide valuable insights based on millions of high-quality, high-coverage SARS-CoV-2 complete protein sequences. Users of this database have the ability to search for information on three amino acid substitution mutation strategies based on gene name, geographical zone, or comparative analysis. Each strategy is presented in five distinct formats which includes: (i) mutated sample frequencies, (ii) heat maps of mutated amino acid positions, (iii) mutation survivals, (iv) natural selections and (v) details of substituted amino acids, including their names, positions, and frequencies. GISAID is a primary database of genomics sequencies of influenza viruses updated daily. SARS2Mutant is a secondary database developed to discover mutation and conserved regions from the primary data to assist with design for targeted vaccine, primer, and drug discoveries.

## INTRODUCTION

The new subfamily member of *Coronavirinae*, subsequently named severe acute respiratory syndrome coronavirus 2 (SARS-CoV-2), caused coronavirus disease 2019 (COVID-19), which appeared for the first time in the Wuhan State of Hubei Province in China, in early December 2019 (1,2). With the worldwide spread of SARS-CoV-2, large populations have been affected, which already accounts for more than 6.1 million deaths and about 493 million cumulative cases globally, as of 8 April 2022 (WHO, Coronavirus (COVID-19) Dashboard, COVID19.who.int). In addition, studies indicate that the numbers of indirect COVID-19 deaths, especially for those who experience multiorgan effects that can involve many body system, including the heart, lung, kidney, and brain, increased rapidly daily (3,4), prompting attention to this disease which has become one of the major treatment priorities of all countries and the World Health Organization (WHO) (2). SARS-CoV-2 is the seventh coronavirus known to infect humans (5) and is classified as a *Sarbecovirus* subgenus, *Betacoronavirus* genus, and *Orthocoronavirus* subfamily member belonging to the *Coronaviridae* family (6). High throughput data techniques such as Next Generation Sequencing (NGS) revealed that this virus shares about 80% genome sequence identity with the severe acute respiratory syndrome coronavirus (SARS-CoV), which emerged in 2002–2003 (7). As of April 2022, almost 10.5 million full genomes are available via the Global Initiative on Sharing All Influenza Data (GISAID), which is one of the main pandemic genome databases.

**Figure 1.**
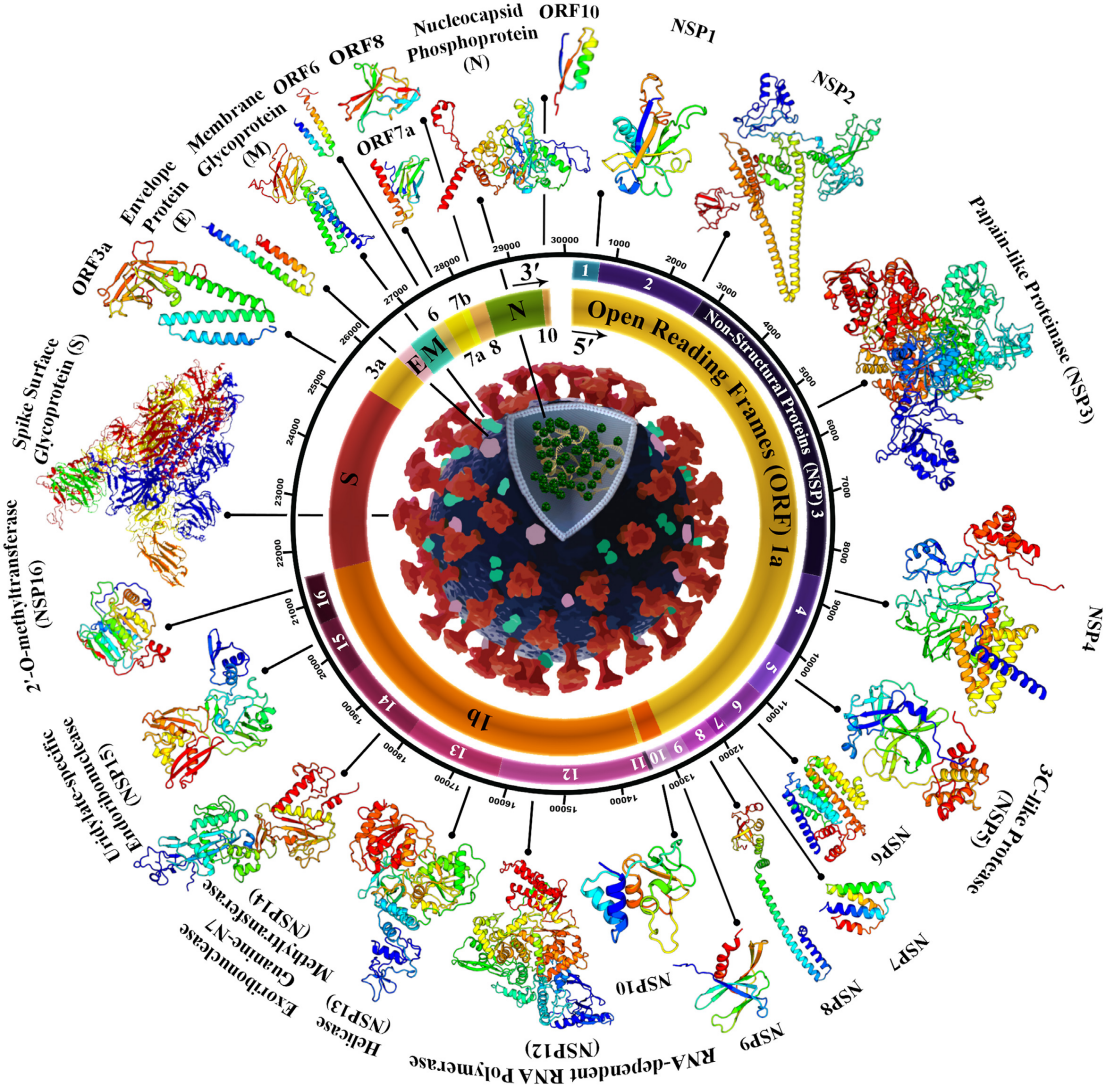
Schematic view of the SARS-CoV-2 particles, genome arrangement, and proteome organization. SARS-CoV-2 is an enveloped positive-sense single-stranded RNA beta coronavirus with a ∼30 kb polycistronic genome that encodes non-structural proteins (ORF1a and ORF1b, that are processed into Nsp1-16) at the 5′-end, and structural proteins (S, E, M and N), and several other accessory factors (ORF3a, 6, 7a, 7b, 8 and 10) at the 3′-end. Wild type of 3D structural models of protein were retrieved from I-TASSER (40).

The SARS-CoV-2 is 50–200 nm in diameter, has a lipid-enveloped, positive sense, and is a single-stranded RNA virus. The full-length RNA genome comprises 29903 nucleotides (nt) consisting of the open reading frames 1a and 1b (ORF1ab). ORF1ab encodes ORF1 polyprotein that are proteolytically processed into 16 mature non-structural proteins (NSPs) that play critical roles as regulatory proteins in viral RNA replication and transcription. Moreover, SARS-CoV-2 contains genes that encode four major structural proteins, including spike surface glycoprotein (S), an envelope protein (E), membrane glycoprotein (M), and nucleocapsid phosphoprotein (N), all of which are responsible for the infectious virion assembly. The N protein packages the RNA genome into a helical ribonucleocapsid. The S, E and M proteins generate the viral envelope. The S protein is comprised of two functional subunits which are involved in viral interactions with a host cell receptor angiotensin-converting enzyme 2 (ACE2) (S1 subunit), as well as mediating the fusion of the host and the viral membrane (S2 subunit). Hence, it is a potential therapeutic target for antiviral drug development. Several other genes translated to proteins called accessory factors are interspersed between structural genes. Although biological functions of these genes have been determined, a number of them continues to remain unclear (7–9). Figure 1 shows an overview of all the details of the wild type of SARS-CoV-2, from the genome to the proteome.

Chronic infection and co-infection of an individual with multiple variants of SARS-CoV-2 and subsequent recombination of the genome play significant roles in the evolution of SARS-CoV-2 variants. It is very important to investigate the diffusion of the new coronavirus variants and the effective methods to contain this spread. Although most of the mutations do not have severe consequences on the spread and mortality rate of the virus, some of them lead to altered disease presentation, increased transmissibility, virulence, or decreased efficacy of diagnostic tests, vaccine-induced protection, and management measures, causing global concern (10–12).

Since early 2020, an abundance of SARS-CoV-2 studies, and several databases have been developed to focus on classification of viruses at the level of genomic mutations and viral evolution (13–15). Despite the great importance of investigating mutations at the amino acid level and their effects on protein function, in order to develop prevention, treatment, and drug discovery (16,17), the available databases reporting single amino acid variations (SAVs) are very limited. The Sars2Mutant database was developed based on SAVs identified in more than ten million complete SARS-CoV-2 genome sequences with high-coverage and high-quality (as of April 2022). The Sars2Mutant database classifies all genes separately by continent, country, and timeline.

This database identifies and analyzes specific positions of mutations at the protein level, classifies mutations by gene frequencies, and provides valuable insights to hotspot positions and highly conserved regions based on alignment to the reference sequence. The Sars2Mutant database offers a user friendly experience, providing transparency of data presentation, and high quality visual illustrations.

## MATERIALS AND METHODS

### Data collection

This inaugural version of the SARS2Mutant database contains 10.5 million high-quality and high-coverage SARS-CoV-2/hCoV-19 protein sequences downloaded from Global Initiative on Sharing Avian Influenza Data (GISAID, https://www.gisaid.org/) (18–20) 24 February 2020 to April 2022.

### Pre-processing and quality control

Sample criteria was selected from human samples labeled with identifiers for time and zone location. Excluded from the sample criteria were non-human samples (such as bat and pangolin), amino-acid sequences (AAs) outside of the reference genome range (Wuhan-WIV04 (EPI_ISL_402124)), samples containing non-specified AAs (reported as X), and samples not reporting geographical location. As of 28 April 2022, more than 10.5 million samples were included in this study, and samples are summarized in Table 1. Table 1 was prepared by following a step by step filtering process using python libraries such as ‘NumPy (V 1.20)’ and ‘Pandas (V 1.5.2)’.

**Table 1.** The number of included samples which sorted step by step

		Criteria trimming			
Gene name	Total	Not match length	Sequence contain X	Non human	Remained
**NSP1**	10261296	586827	433559	7298	9233612
**NSP2**	10280285	23067	940384	5712	9311122
**NSP3**	10285623	2240262	1971864	3322	6070175
**NSP4**	10283271	18737	1357844	7135	8,899555
**NSP5**	10283326	13584	650306	7390	9612046
**NSP6**	10283424	4573308	556800	4278	5149038
**NSP7**	10283211	1831	113086	7955	10160339
**NSP8**	10283099	2993	321269	7907	9950930
**NSP9**	10283166	2303	171536	7942	10101385
**NSP10**	10283220	5178	386807	7783	9883452
**NSP11**	10282621	406	248550	8011	10025654
**NSP12**	10283038	15751	1417103	6870	8843314
**NSP13**	10283161	8417	947020	7320	9320404
**NSP14**	10283125	16466	1963818	6608	8296233
**NSP15**	10283011	11275	1082988	5821	9182927
**NSP16**	10284335	14175	987187	6527	9276446
**ORF3a**	10285527	218561	582697	7266	9477003
**ORF6**	10282433	142987	132233	7818	9999395
**ORF7a**	10282309	732602	496921	7595	9045191
**ORF7b**	10269432	477420	491389	7753	9292870
**ORF8**	10278801	5582221	495547	4480	4196553
**ORF9b**	10282579	3215630	227157	5499	6834293
**ORF9c**	10280158	485696	156699	7228	9630535
**S**	10391577	8545727	894375	1015	9504,60
**E**	10284323	1,97859	163922	8012	9914530
**M**	10284401	94574	1322197	7166	8860464
**N**	10283011	3265363	647251	4939	6365458

### Variants calling and functional annotation

Sequence alignments were made by detecting total single amino-acid (AA) variations against the reference genome, Wuhan-WIV04 (EPI_ISL_402124). Wuhan-WIV04 genome is the full-length protein sequence reference of the SARS-CoV-2 identified in China in December 2019, known as the reference sequence in GISAID that determined each gene location precisely. Another reference sequence reported in NCBI is Wuhan-WIV04 (NCBI: NC 045512.2), one of these sequences, Wuhan-WIV04 and Wuhan-Hu1, are the same in protein levels. At the genome levels, Wuhan-Hu-1 has 12 more poly-A tails at the end of the RNA genome, but protein levels are unaffected. All SARS-CoV-2 sequence mutations are scheduled to be updated monthly and powered by the GISAID database.

The complete reference sequences of SARS-CoV-2 were captured from the GISAID database. Access to this database is by permission of John A. Burns School of Medicine Department of Quantitative Health Sciences, and data is preprocessed with Python libraries. After filtering low-quality samples and removing white spaces within the series, we designed a unique library based on an exact match algorithm pairwise to align SARS-CoV-2 sequences with the reference genome Wuhan-WIV04. This library is available on https://github.com/karimrahimian/covid_analysis, which can handle the big data sequence pairwise aligner that aligns long protein sequences in the FASTA format based on our strategy for analyzing whole SARS-CoV-2 sequences. Each sequence was aligned separately with the reference genome, and the variations were reported. The pairwise alignment results are not affected by other sequences. The worldwide mutation rate for each gene was obtained by dividing the number of identified mutations by the number of gene samples in the ‘Remained’ column of Table 1.

To report the rate of mutations for each continent, the same method was used, dividing the total mutation number of the specific gene by the number of gene samples in the ‘Remained’ column of Table 1.

The SARS2Mutant database is specialized for identifying protein mutations, the details of each mutation, including the exact location, its geographical incidence, and the replaced AA. Therefore, each sequence is labeled, and an identification profile for classification data is created. The data structure can search and categorize data based on the mutant ratio in each part of the protein sequence (NSP1-NSP16, S, E, M, N, accessory factors), the concurrence, and mutation frequency by geographical zone.

### Platform architectural design and structure

**Figure 2.**
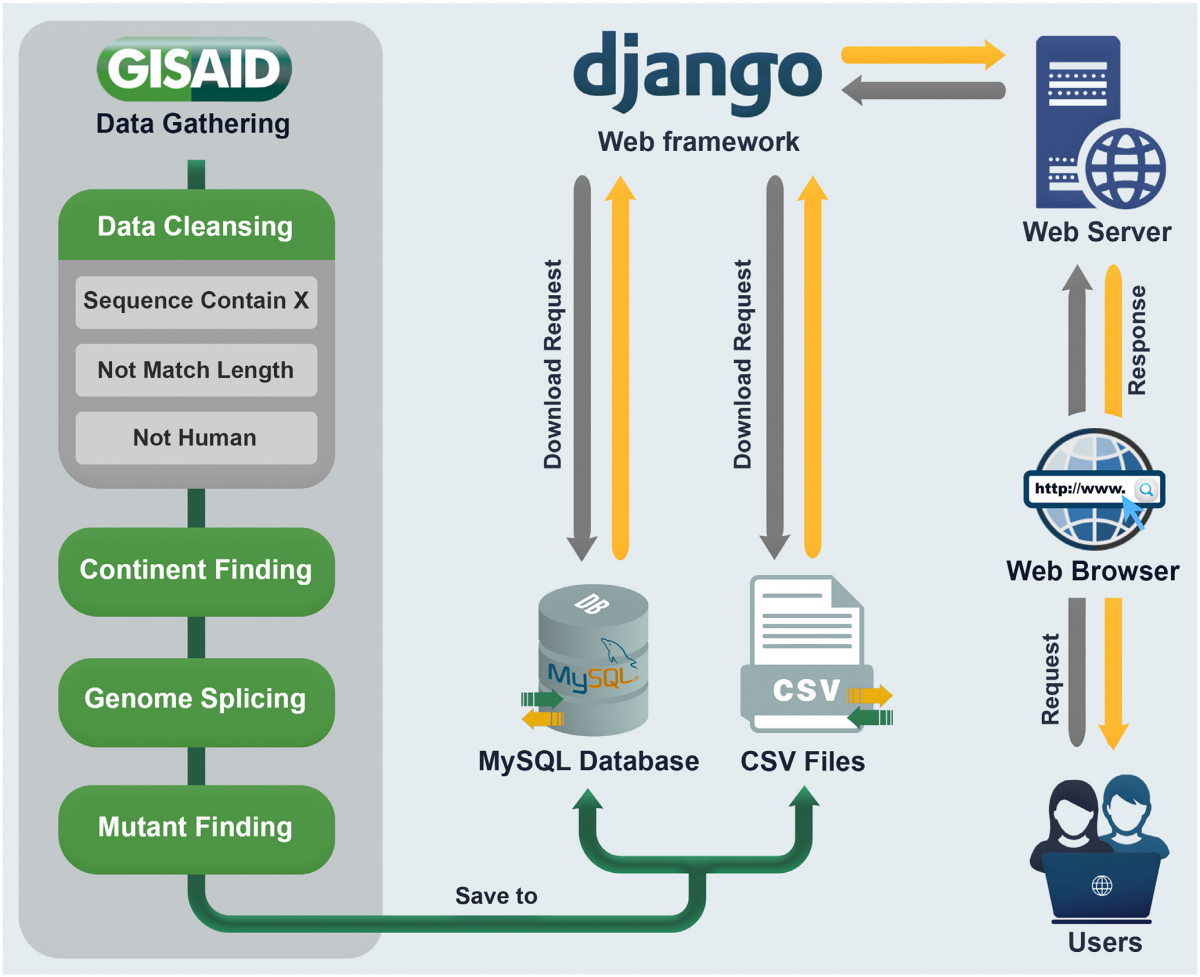
Database architecture and workflow. Request mechanisms from users to the database.

The SARS2Mutant web platform and relational database connection were implemented using Django package in Python 3.9.7 programming environment for the backend. HTML, CLS and JavaScript were used to design the frontend. An object-oriented architecture was designed and implemented in a relational database (MySQL) to store the annotated variants instead of the conventional spreadsheet file (CSV/VCF) to allow further flexibility when formulating search queries and alleviate database load by reducing data duplication that causes reduced data load time. The database architecture and relationships between tables are shown in Figure 2. The Python-based Django framework with a monolithic architectural design was employed in the development of our project. The project comprises of 15 tables, including tables to store genes, regions, pies, heat-maps, timelines, stacks, user information, visitor information and site details. Complete details about the MySQL tables, including their respective names, column types, primary key fields, and relationships, have been provided in Supplementary A. Additionally, the entity-relationship diagram illustrates the interrelation between the tables.

## RESULTS

**Figure 3.**
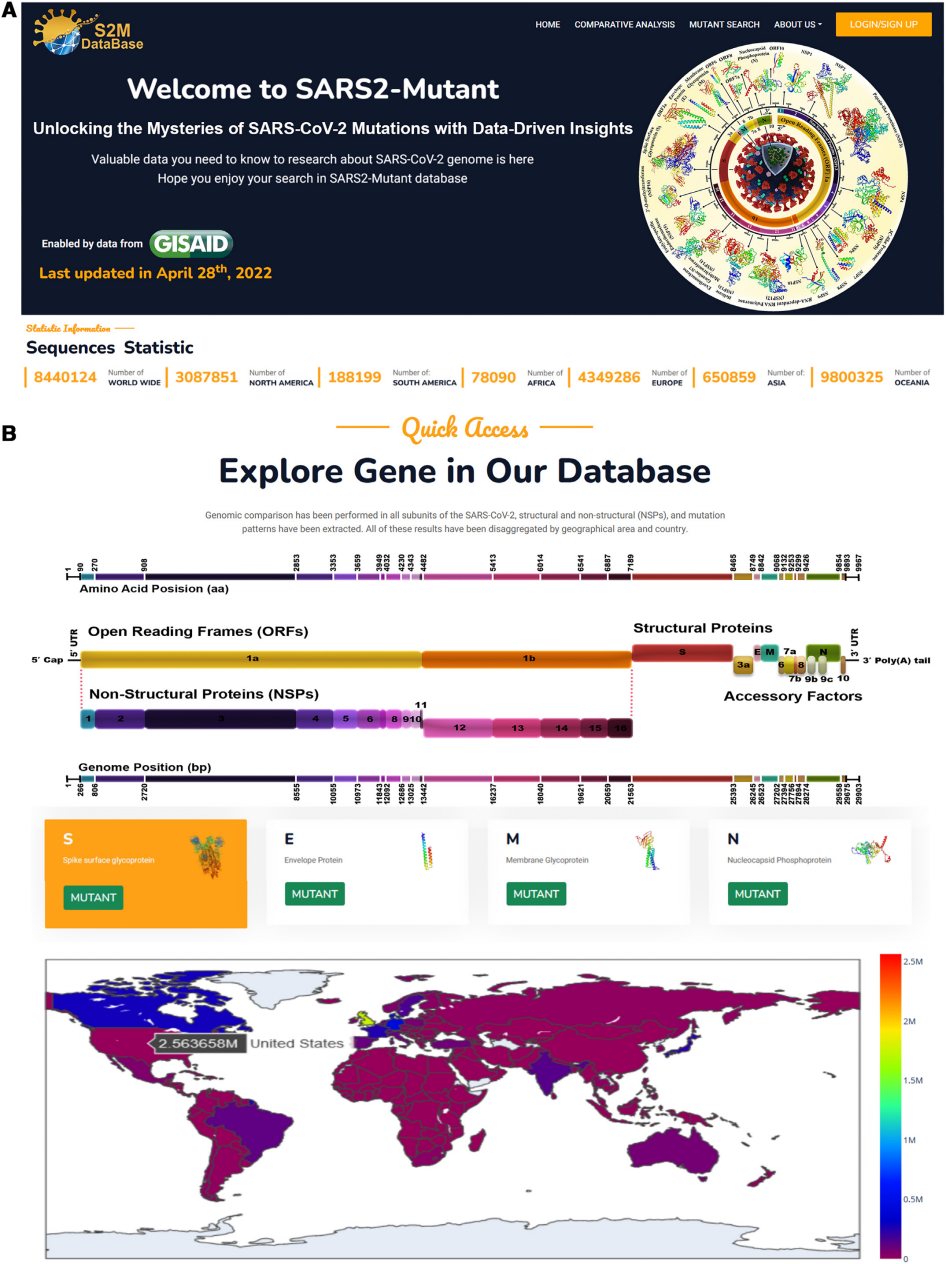
Database structure. (**A**) The homepage, services and facilities. (**B**) The quick access part of the database that helps the user find the results quickly and clearly.

The SARS2Mutant database homepage offers a comprehensive overview of the site's design goals and principles, as depicted in Figure 3A. To facilitate the formulation of research ideas, the database includes a table of SARS-CoV-2 genes that succinctly summarizes their functions, thereby enhancing the interpretation of results.

The SARS2Mutant taskbar contains five tabs, namely ‘Home,’ ‘Comparative Analysis,’ ‘Mutant Search,’ ‘About Team’ and ‘Login/Sign Up.’ Tabs 1, 2 and 3 correspond to three primary search strategies, namely ‘Comparative Analysis’, ‘Mutant Search and 'Quick Access’.

The ‘Quick Access’ section is located at the top of the site and comprises 28 cards with quick access links. By hovering the mouse over each card, the user can transfer the selected analysis to the results page, which is presented in two categories for review.

The first category, ‘Top 100 mutation reports’, provides mutation occurrence distributions, AAs mutation position, substitution AA, hotspot maps, and top 10 sustainable monthly tracking timelines. Access to the entire dataset requires user registration, which allows the downloading CSV files for reports beyond the first 100 mutations.

The second category features top mutation selections based on the occurrence frequency from different geographical locations, which are mapped onto a global graph. Overall, the SARS2Mutant database is designed to provide a user-friendly platform that enhances the accessibility and interpretability of SARS-CoV-2 mutation data.

The ‘Quick access’ section also allows users faster and easier access to the target gene of interest (Figure 3B). At the bottom of the homepage is an interactive ‘Worldwide Mutation Distribution.’ This section allows users to find more categorized information about mutation frequency in the countries/areas by moving the mouse over the maps to any part of their area of interest. A table within the ‘Worldwide Mutation Distribution’ tab shows the counts of SAVs in each country/area. A case study was conducted on NSP1 mutation virulence and natural selection including evolutionary trends from six continents (21). This publication represents an examination of the potential applications of available data within the present database and expands researchers understanding of the tracking of mutations across different geographical regions.

### Protein name search, ‘quick access’

The ‘*Protein Name search*’ allows users to explore SAVs in a particular region of the viral-specific protein (‘*Protein-based search*’). The search shows mutation frequency among samples, the comparative analysis between hotspot versus conserved region in the referenced gene, substituted AAs name and frequency, monthly mutation survival trend and geographical mutations distribution. SAVs within the selected proteins are presented as visualizations of pie chart, heatmap, stacked plot, timeline chart, and worldwide map.

**Figure 4.**
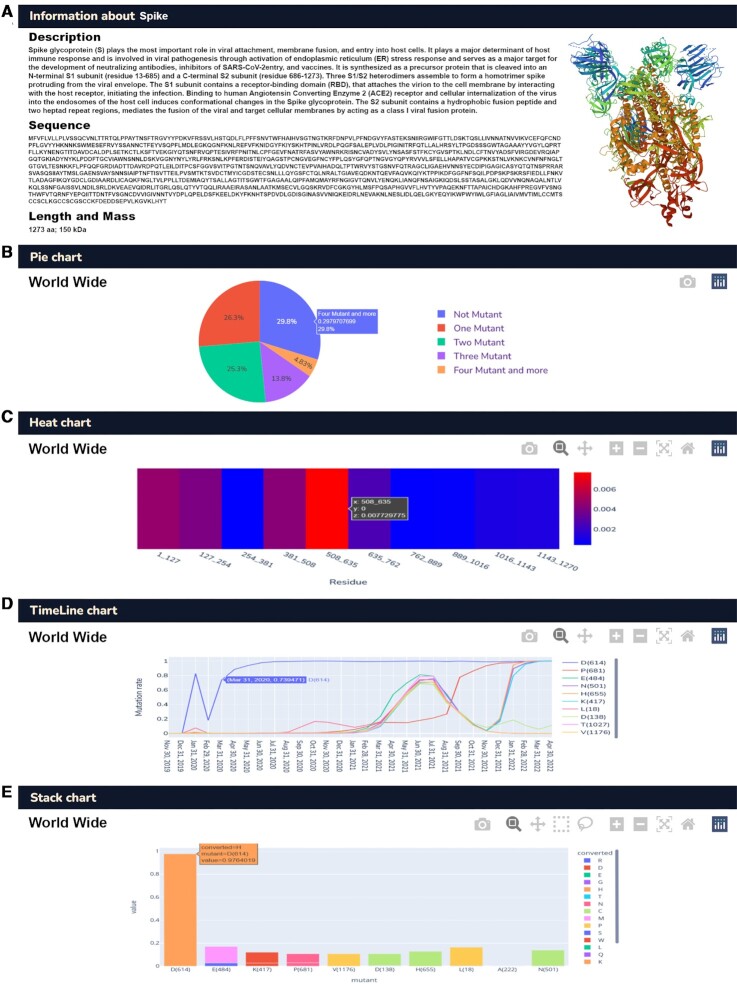
Database data representation structure. (**A**) Protein description, functions, and official sequence. (**B**) mutation frequency among all analyzed samples. (**C**) Hotspot genome positions versus conserved. (**D**) Mutation detection trend during the month. (**E**) Mutation frequencies and Substitution amino acids.

For example, to find the spike protein (S) mutations, in part A, there is a description of protein activity and its official sequence (Figure 4A). Part B pie chart shows that 4.82%, 26.31%, 25.31%, 13.75% and 29.79% of samples are conserved, with one mutation, two mutations, and three mutations and more than four mutations, respectively, worldwide (Figure 4B). Part C heatmap shows the total length of each gene divided into ten sections where the x-axis represents the hotspot positions. The *y*-axis expresses the mutation frequency of each position. In the spike example, the most frequent mutation in the gene occurs at the 508–635 AA positions (Figure 4C).

Part D, worldwide timeline chart represents the mutation point position of mutations depicted by colors. Based on our outcomes, the chart shows that the D614 position mutation was observed for the first time in December 2019, and the mutation numbers increased after February 2020. The second highest mutation frequency, E484, shows an uptrend beginning from November 2021 to the present. The timeline chart shows the sustainability of each mutation during the month. The *y*-axis shows the mutation rate, and the *x*-axis represents each month (Figure 4D).

In Part E, the worldwide map was designed to represent the distribution of the specific mutations globally, by tracking the AA changes. This includes type and frequency in each position where the y-axis of the stacked plot shows the Log frequency for comparison, and the x-axis is the name of wild-type AAs and their position. The case study example of D614 detected Glycine (D614G) with the highest frequency of AA substitution, and asparagine (D614N) with the second highest (Figure 4E).

As a result, each mutation based on evolutionary parameters during the pandemic showed different survival patterns and frequency of AA changes. It could indicate which positions could play an essential role in that specific gene function, which could be more adaptable to different situations. It is notable that in all the mentioned graphs and maps, users are allowed to find more categorized information about mutation frequency in the countries/areas by moving the mouse over the maps to any part of their specific interest or by free registration for database access to the total data, representing more than 100 mutations.

### SAVs first geographic identification query, ‘mutant search’

**Figure 5.**
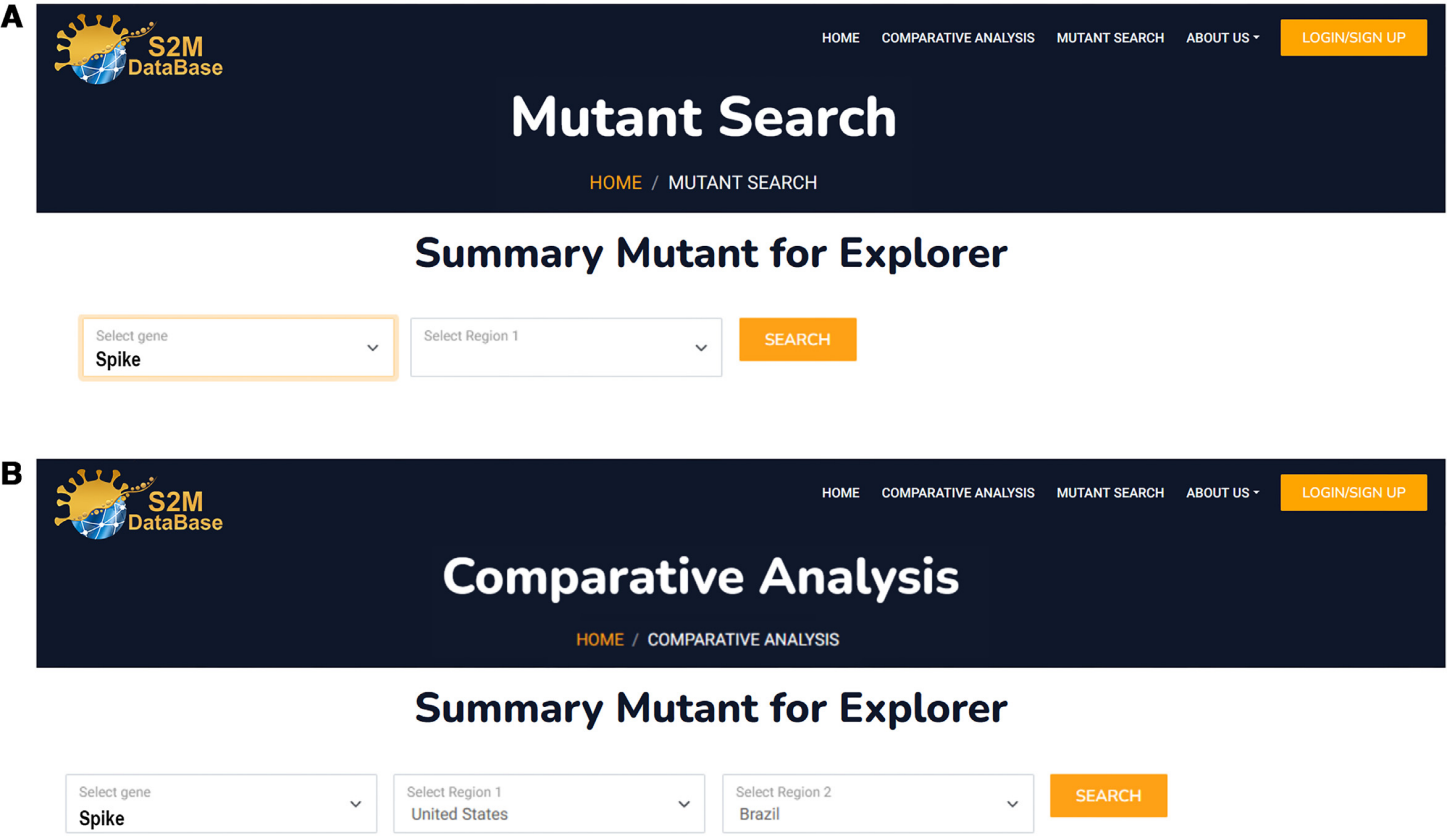
Database search strategy. (**A**) Mutant Search allows the user to search based on the candidate gene. (**B**) Comparative analysis enables user to search and compare the candidate gene between two different areas.

Similar to other viruses, SARS-CoV-2 has created genetic diversity via temporally accumulated mutations. The ‘SAV first geographic identification query’ is set up for users to overview the geographical zones where SAVs are discovered and the exact AA position and substitution AA. This means that the area where the mutation was observed is shown by selecting particular mutations (Figure 5A).

### Search mutations based on geographical zone, ‘comparative analysis’

The ‘Continent/ Country search’ helps users focus on SAVs identified in a specific continent or Country (‘Region-based search’).When comparing two countries, the summary of mutations includes country names, positions of SAVs, numbers and percentages of viral genomes carrying the SAV, and substituted AAs reported in the selected area.

In addition, users can also filter or do a quick search on SAVs given attractive attributes by adding the keyword in the search box. Users can also select the gene of interest and target the country/continent to compare the results between the two regions. The results are formatted in the same structures as in the ‘Quick Search ’ section, and SAVs within selected areas are presented in the same graphs. All data is available to download in CSV file format (Figure 5B).

## DISCUSSION

Numerous online databases of SARS-CoV-2 mutations have been developed over the last two years (22–26). The remarkable difference between the SARS2Mutant database and similar available databases such as GESS [29], Covariants [17], COVMT[15], VirusViz [16] and IDBSV [30] is simply that data visualization over thousands of annotated records covers complete SARS-CoV-2 genome, and other numerous parameters summarized and compared in Table 2. The SARS2Mutant database provides a user-friendly environment for easy operations to obtain a holistic overview of SARS-CoV-2 SAVs.

**Table 2.** Comparison checklist between same concept database with SARS2mutant

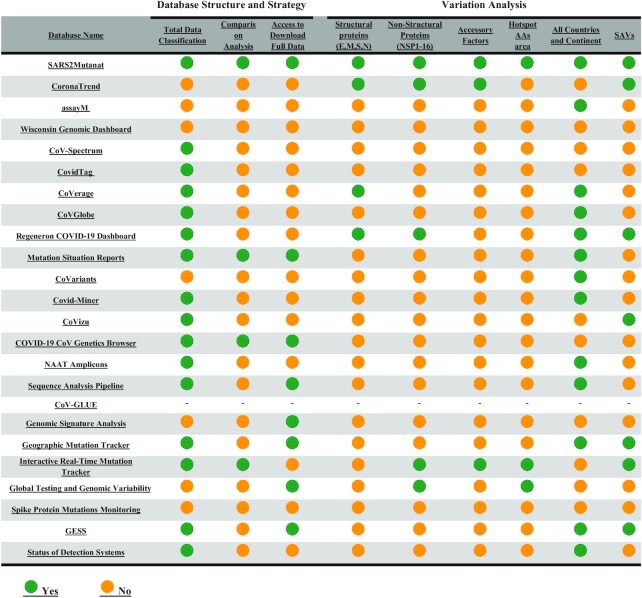

Each protein has a specific profile and includes classified data that can be compared with other proteins. Additionally, according to a study on the protein level, only important mutations which appear at the phenotype level are reported. Silent mutations which do not result in a change in the AAS and subsequently have no effect on protein folding and function are not considered in the reports. In addition, according to the SARS-CoV-2 gene structure, the exact location of mutations in each gene was reported separately by the SARS2Mutant database, which will be very effective in investigating drug and vaccine effects. Researchers could search for specific mutations based on proteins, AA location, and selective substitution AA. Since proteins play a role in the presence of phenotypes, the accumulation of various mutations at the level of AAs can change the structure of the proteins and ultimately produce a new phenotype characterization that can give a new feature to the mutant virus or alter the impact on its pathogenesis and virulence (27,28).

The evolution of viruses depends on the co-occurrence of various mutations in multiple genes or within a single gene. The accumulation of virus mutations has resulted in drug-resistant or vaccine-escape mutants, leading to a continuous need to design new drugs or vaccines (29). Ongoing screening of functional mutations will help provide insight into the evolution and genetic diversity of SARS-CoV-2, which is also critical for developing efficient antiviral drugs or vaccines against this virus (30). Vaccination, ANTICOV therapeutic strategies, and diagnostic products are potent tools for inhibiting global disease spreads. Extensive global research and efforts have been dedicated to developing an efficient vaccine to elicit a rapid and robust immune-protective response, and provide solid long-lasting immunity against different strains of SARS-CoV-2 (31).

On the one hand, the ideal vaccine must have strong immunogenicity. It should be able to elicit targeted humoral and cellular immune responses via specific epitopes. On the other hand, it should also be less immunotoxin and non-allergenic (31–33). Moreover, to provide a universal antibody to neutralize different viral strains, the conservation of targeted sequences, such as the T and B cell epitopes that elicit cellular and humoral immune responses, must also be considered. The SARS2Mutant database can greatly help calibrate mutation rates for specially designed regions.

The dynamics of functional mutations which can give rise to drug resistance presents a challenge for us to understand and manage the mechanisms of SARS-CoV-2 drug resistance, which is imperative to find viable drug target candidates for designing effective antiviral drugs (34–36). Also, functional mutations can impact the results of diagnostic tests and lead to false negative using rapid antigen tests. Therefore, it is essential to monitor the SARS-CoV-2 mutations to develop stable and reliable diagnostic tests (37–39). Accordingly, mutation screening investigations in a fast, reliable, and cost-effective way with the implementation of databases will help develop effective coping strategies during the COVID-19 pandemic.

According to GISAID powered databases that used the same data representing different aspects, we summarized the difference between SARS2Mutant database and others in Figure 4. Technical structures and data representations are evaluated and compared based on two main parts of the database, ‘Mutant Search’ and ‘Comparative analysis’. Although SARS2Mutant database's primary goal is to present molecular details, it also shows the epidemiological status summarized in Figure 4. The homepage of SARS2Mutant contains information about the number of samples collected. Search functions on SARS2Mutant offer users more considerable flexibility to browse and search SAV patterns from different aspects, such as protein locations, sample geographical zone, and mutation frequency rates, while focusing on SAV characteristics. A critical feature of SARS2Mutant is using the correlation function on SAVs. A parameter, concurrence ratio R, is adopted to identify simultaneous SAVs. SARS2Mutant database also provides a novel process for SAV original query to monitor newly emerged SAVs each month. Through visualized distributions and graphical diagrams, it helps users better understand the migration, transmission, spread, and evolution trend of SARS-CoV-2.

The goal of SARS2Mutant is to provide a user-friendly database to explore the associations and interactions between SAVs. In general, by fetching the data of SAVs and using functions embedded in SARS2Mutant to analyze their significant features, users may gain new insights into the molecular drivers of SARS-CoV-2 transmission, migration, and evolution.

With the aim of enhancing public safety across nations and mitigating the impact of the virus, we propose augmenting the quantity of virus sequences obtained from each country to enable comprehensive analysis of the data within national boundaries. This approach will facilitate development of tailored vaccination and treatment programs specific to individual countries which customized treatment and vaccination strategies, informed by the genetic and blueprint characteristics of the virus prevalent in each region.

As of 28 April 2022, SARS2Mutant hosted over sixty thousand variations extracted from the analysis of over 10.5 million SARS-CoV-2 samples. Notably, the analysis of these mutations revealed consistent results with the findings from specialized studies and literature.

## DATA AVAILABILITY

GISAID data provided on SARS2Mutant database are subject to GISAID’S terms and conditions (https://www.gisaid.org/registration/terms-of-use/).

All used library and aligner codes are available on https://github.com/karimrahimian/covid_analysis.

Database structure and codes are available on https://github.com/karimrahimian/sars2mutant/tree/main and https://doi.org/10.5281/zenodo.7783399.

## Supplementary Material

lqad037_Supplemental_File
