# Supplementary material for: SARS2Mutant: SARS-CoV-2 amino-acid mutation atlas database
Source: NAR Genom Bioinform. 2023 Apr 24;5(2):lqad037. doi: 10.1093/nargab/lqad037 (PMC10124966; doi:10.1093/nargab/lqad037)
Supplement: lqad037_Supplemental_File [file lqad037_supplemental_file.pdf]

Table of contents

1 auth_group	Page number: 2
2 auth_group_permissions	Page number: 3
3 auth_permission	Page number: 4
4 django_admin_log	Page number: 5
5 django_content_type	Page number: 6
6 django_migrations	Page number: 7
7 django_session	Page number: 8
8 website_country	Page number: 9
9 website_gene	Page number: 10
10 website_geneinfo	Page number: 11
11 website_heatmap	Page number: 12
12 website_mutant	Page number: 13
13 website_mutantinfo	Page number: 14
14 website_mutantinfocountry	Page number: 15
15 website_news	Page number: 16
16 website_pie	Page number: 17
17 website_published	Page number: 18
18 website_region	Page number: 19
19 website_registeruser	Page number: 20
20 website_registeruser_groups	Page number: 21
21 website_registeruser_user_permissions	Page number: 22
22 website_stack	Page number: 23
23 website_stacknew	Page number: 24
24 website_timeline	Page number: 25
25 website_visitor	Page number: 26
26 Relational schema	Page number: 27

1 auth_group

Creation: Sep 25, 2022 at 09:35 PM

Column	Type	Attributes	Null	Default	Extra	Links to	Comments	MIME
id	int(11)		No		auto_increment			
name	varchar(150<br>)		No					

2 auth_group_permissions

Creation: Sep 25, 2022 at 09:35 PM

Column	Type	Attributes	Null	Default	Extra	Links to	Comments	MIME
id	bigint(20)		No		auto_increment			
group_id	int(11)		No			-> auth_group.id<br>ON UPDATE RESTRICT<br>ON DELETE RESTRICT		
permission_id	int(11)		No			-> auth_permission.id<br>ON UPDATE RESTRICT<br>ON DELETE RESTRICT		

3 auth_permission

Creation: Sep 25, 2022 at 09:35 PM

Column	Type	Attributes	Null	Default	Extra	Links to	Comments	MIME
id	int(11)		No		auto_increment			
name	varchar(255<br>)		No					
content_type_id	int(11)		No			-> django_content_type.id<br>ON UPDATE RESTRICT<br>ON DELETE RESTRICT		
codename	varchar(100<br>)		No					

4 django_admin_log

Creation: Sep 25, 2022 at 09:35 PM

Column	Type	Attributes	Null	Default	Extra	Links to	Comments	MIME
id	int(11)		No		auto_increment			
action_time	datetime(6)		No					
object_id	longtext		Yes	NULL				
object_repr	varchar(200)		No					
action_flag	smallint(5)	UNSIGNED	No					
change_message	longtext		No					
content_type_id	int(11)		Yes	NULL		-> django_content_type.id ON UPDATE RESTRICT ON DELETE RESTRICT		
user_id	bigint(20)		No			-> website_registeruser.id ON UPDATE RESTRICT ON DELETE RESTRICT		

5 django_content_type

Creation: Sep 25, 2022 at 09:35 PM

Column	Type	Attributes	Null	Default	Extra	Links to	Comments	MIME
id	int(11)		No		auto_increment			
app_label	varchar(100<br>)		No					
model	varchar(100<br>)		No					

6 django_migrations

Creation: Sep 25, 2022 at 09:35 PM

Column	Type	Attributes	Null	Default	Extra	Links to	Comments	MIME
id	bigint(20)		No		auto_increment			
app	varchar(255<br>)		No					
name	varchar(255<br>)		No					
applied	datetime(6)		No					

7 django_session

Creation: Sep 25, 2022 at 09:35 PM

Column	Type	Attributes	Null	Default	Extra	Links to	Comments	MIME
session_key	varchar(40)		No					
session_data	longtext		No					
expire_date	datetime(6)		No					

8 website_country

Creation: Sep 25, 2022 at 09:35 PM

Column	Type	Attributes	Null	Default	Extra	Links to	Comments	MIME
id	bigint(20)		No		auto_increment			
country_name	varchar(100<br>)		No					
sequence_count	int(11)		No					
group	varchar(5)		No					
region_id	bigint(20)		No			-> website_region.id<br>ON UPDATE RESTRICT<br>ON DELETE RESTRICT		

9 website_gene

Creation: Sep 25, 2022 at 09:35 PM

Column	Type	Attributes	Null	Default	Extra	Links to	Comments	MIME
id	bigint(20)		No		auto_increment			
name	varchar(20)		No					
title	longtext		No					
category	varchar(10)		No					
alias_name	varchar(20)		No					

10 website_geneinfo

Creation: Sep 25, 2022 at 09:35 PM

Column	Type	Attributes	Null	Default	Extra	Links to	Comments	MIME
id	bigint(20)		No		auto_increment			
length	int(11)		No					
sequence	longtext		No					
features	longtext		No					
description	longtext		No					
gene_id	bigint(20)		No			-> website_gene.id<br>ON UPDATE RESTRICT<br>ON DELETE RESTRICT		

11 website_heatmap

Creation: Sep 25, 2022 at 09:35 PM

Column	Type	Attributes	Null	Default	Extra	Links to	Comments	MIME
id	bigint(20)		No		auto_increment			
range	varchar(30)		No					
value	double		No					
country_id	bigint(20)		No			-> website_country.id<br>ON UPDATE RESTRICT<br>ON DELETE RESTRICT		
gene_id	bigint(20)		No			-> website_gene.id<br>ON UPDATE RESTRICT<br>ON DELETE RESTRICT		

12 website_mutant

Creation: Sep 25, 2022 at 09:35 PM

Column	Type	Attributes	Null	Default	Extra	Links to	Comments	MIME
id	bigint(20)		No		auto_increment			
amino_acid	varchar(1)		No					
position	int(11)		No					
mutant	longtext		No					
gene_id	bigint(20)		No			-> website_gene.id<br>ON UPDATE RESTRICT<br>ON DELETE RESTRICT		

13 website_mutantinfo

Creation: Sep 25, 2022 at 09:35 PM

Column	Type	Attributes	Null	Default	Extra	Links to	Comments	MIME
id	bigint(20)		No		auto_increment			
sequence_id	varchar(50)		No					
date	date		No					
country_id	bigint(20)		No			-> website_country.id<br>ON UPDATE RESTRICT<br>ON DELETE RESTRICT		
mutant_id	bigint(20)		No			-> website_mutant.id<br>ON UPDATE RESTRICT<br>ON DELETE RESTRICT		

14 website_mutantinfocountry

Creation: Sep 25, 2022 at 09:35 PM

Column	Type	Attributes	Null	Default	Extra	Links to	Comments	MIME
id	bigint(20)		No		auto_increment			
country_id	bigint(20)		No			-> website_country.id<br>ON UPDATE RESTRICT<br>ON DELETE RESTRICT		
mutant_id	bigint(20)		No			-> website_mutant.id<br>ON UPDATE RESTRICT<br>ON DELETE RESTRICT		
frequency	double		No					

15 website_news

Creation: Sep 25, 2022 at 09:35 PM

Column	Type	Attributes	Null	Default	Extra	Links to	Comments	MIME
id	bigint(20)		No		auto_increment			
title	longtext		No					
image	varchar(100)		No					

16 website_pie

Creation: Sep 25, 2022 at 09:35 PM

Column	Type	Attributes	Null	Default	Extra	Links to	Comments	MIME
id	bigint(20)		No		auto_increment			
range	varchar(30)		No					
value	double		No					
country_id	bigint(20)		No			-> website_country.id<br>ON UPDATE RESTRICT<br>ON DELETE RESTRICT		
gene_id	bigint(20)		No			-> website_gene.id<br>ON UPDATE RESTRICT<br>ON DELETE RESTRICT		

17 website_published

Creation: Sep 25, 2022 at 09:35 PM

Column	Type	Attributes	Null	Default	Extra	Links to	Comments	MIME
id	bigint(20)		No		auto_increment			
title	varchar(500<br>)		No					

18 website_region

Creation: Sep 25, 2022 at 09:35 PM

Column	Type	Attributes	Null	Default	Extra	Links to	Comments	MIME
id	bigint(20)		No		auto_increment			
region_name	varchar(20)		No					

19 website_registeruser

Creation: Sep 25, 2022 at 09:35 PM

Column	Type	Attributes	Null	Default	Extra	Links to	Comments	MIME
id	bigint(20)		No		auto_increment			
last_login	datetime(6)		Yes	NULL				
is_superuser	tinyint(1)		No					
full_name	varchar(30)		No					
email	varchar(40)		No					
grant	tinyint(1)		No					
university	varchar(50)		No					
password	longtext		No					
is_staff	tinyint(1)		No					
is_activate	tinyint(1)		No					

20 website_registeruser_groups

Creation: Sep 25, 2022 at 09:35 PM

Column	Type	Attributes	Null	Default	Extra	Links to	Comments	MIME
id	bigint(20)		No		auto_increment			
registeruser_id	bigint(20)		No			-> website_registeruser.id<br>ON UPDATE RESTRICT<br>ON DELETE RESTRICT		
group_id	int(11)		No			-> auth_group.id<br>ON UPDATE RESTRICT<br>ON DELETE RESTRICT		

21 website_registeruser_user_permissions

Creation: Sep 25, 2022 at 09:35 PM

Column	Type	Attributes	Null	Default	Extra	Links to	Comments	MIME
id	bigint(20)		No		auto_increment			
registeruser_id	bigint(20)		No			-> website_registeruser.id<br>ON UPDATE RESTRICT<br>ON DELETE RESTRICT		
permission_id	int(11)		No			-> auth_permission.id<br>ON UPDATE RESTRICT<br>ON DELETE RESTRICT		

22 website_stack

Creation: Sep 25, 2022 at 09:35 PM

Column	Type	Attributes	Null	Default	Extra	Links to	Comments	MIME
id	bigint(20)		No		auto_increment			
mutant	varchar(30)		No					
value	double		No					
A	double		No					
R	double		No					
N	double		No					
D	double		No					
C	double		No					
E	double		No					
Q	double		No					
G	double		No					
H	double		No					
I	double		No					
L	double		No					
K	double		No					
M	double		No					
F	double		No					
P	double		No					
S	double		No					
T	double		No					
W	double		No					
Y	double		No					
V	double		No					
country_id	bigint(20)		No			-> website_country.id ON UPDATE RESTRICT ON DELETE RESTRICT		
gene_id	bigint(20)		No			-> website_gene.id ON UPDATE RESTRICT ON DELETE RESTRICT		

23 website_stacknew

Creation: Sep 25, 2022 at 09:35 PM

Column	Type	Attributes	Null	Default	Extra	Links to	Comments	MIME
id	bigint(20)		No		auto_increment			
mutant	varchar(30)		No					
value	double		No					
converted	varchar(1)		No					
country_id	bigint(20)		No			-> website_country.id<br>ON UPDATE RESTRICT<br>ON DELETE RESTRICT		
gene_id	bigint(20)		No			-> website_gene.id<br>ON UPDATE RESTRICT<br>ON DELETE RESTRICT		

24 website_timeline

Creation: Sep 25, 2022 at 09:35 PM

Column	Type	Attributes	Null	Default	Extra	Links to	Comments	MIME
id	bigint(20)		No		auto_increment			
date	date		No					
mutant	varchar(30)		No					
value	double		No					
country_id	bigint(20)		No			-> website_country.id<br>ON UPDATE RESTRICT<br>ON DELETE RESTRICT		
gene_id	bigint(20)		No			-> website_gene.id<br>ON UPDATE RESTRICT<br>ON DELETE RESTRICT		

25 website_visitor

Creation: Sep 25, 2022 at 09:35 PM

Column	Type	Attributes	Null	Default	Extra	Links to	Comments	MIME
id	bigint(20)		No		auto_increment			
ip_addresses	varchar(16)		No					

PDF export page

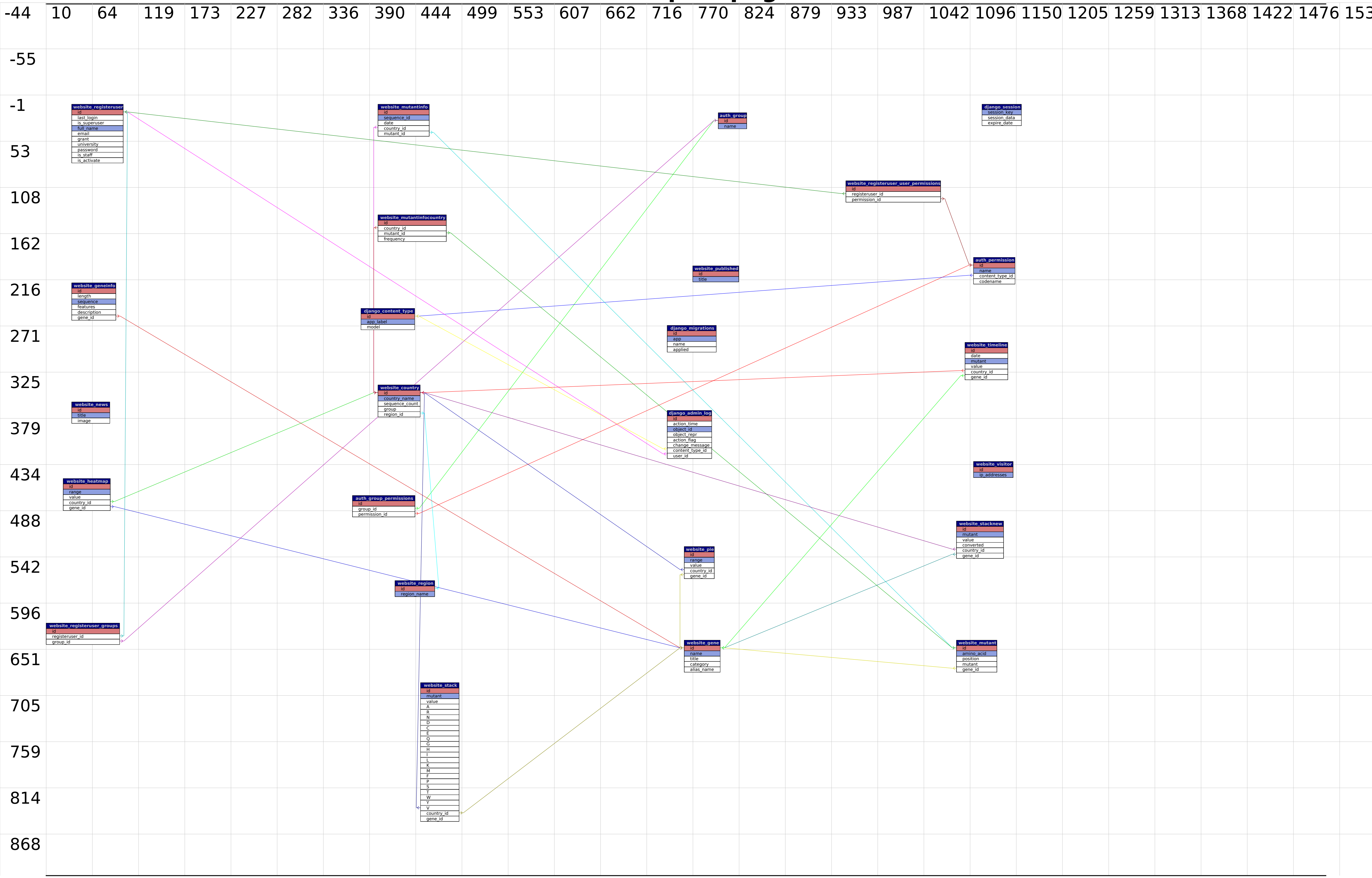

 Feb 10, 2023 at 12:14 AM
